# Bioleaching Modeling—A Review

**DOI:** 10.3390/ma16103812

**Published:** 2023-05-18

**Authors:** Manuel Saldaña, Matías Jeldres, Felipe M. Galleguillos Madrid, Sandra Gallegos, Iván Salazar, Pedro Robles, Norman Toro

**Affiliations:** 1 Faculty of Engineering and Architecture, Arturo Prat University, Iquique 1110939, Chile; chichined@gmail.com (S.G.); notoro@unap.cl (N.T.); 2 Departamento de Ingeniería Química y Procesos de Minerales, Universidad de Antofagasta, Antofagasta 1270300, Chile; hugo.jeldres.valenzuela@ua.cl; 3 Centro de Desarrollo Energético Antofagasta, Universidad de Antofagasta, Antofagasta 1271155, Chile; felipe.galleguillos@uantof.cl; 4 Departamento de Ingeniería Civil, Universidad Católica del Norte, Antofagasta 1270709, Chile; isalazar@ucn.cl; 5 Escuela de Ingeniería Química, Pontificia Universidad Católica de Valparaíso, Valparaíso 2340000, Chile; pedro.robles@pucv.cl

**Keywords:** mineral bioleaching, mineral processing, microorganisms, theoretical and empirical modeling, machine learning

## Abstract

The leaching of minerals is one of the main unit operations in the metal dissolution process, and in turn it is a process that generates fewer environmental liabilities compared to pyrometallurgical processes. As an alternative to conventional leaching methods, the use of microorganisms in mineral treatment processes has become widespread in recent decades, due to advantages such as the non-production of emissions or pollution, energy savings, low process costs, products compatible with the environment, and increases in the benefit of low-grade mining deposits. The purpose of this work is to introduce the theoretical foundations associated with modeling the process of bioleaching, mainly the modeling of mineral recovery rates. The different models are collected from models based on conventional leaching dynamics modeling, based on the shrinking core model, where the oxidation process is controlled by diffusion, chemically, or by film diffusion until bioleaching models based on statistical analysis are presented, such as the surface response methodology or the application of machine learning algorithms. Although bioleaching modeling (independent of modeling techniques) of industrial (or large-scale mined) minerals is a fairly developed area, bioleaching modeling applied to rare earth elements is a field with great growth potential in the coming years, as in general bioleaching has the potential to be a more sustainable and environmentally friendly mining method than traditional mining methods.

## 1. Introduction

In recent times, the global production of mining industries has experienced a decline due to temporary suspensions of smelters for maintenance and updates, caused by health contingencies in various countries, such as sustained drops in ore grades from mineral deposits [1]. There are different processes for treating or extracting valuable minerals from those without economic value, such as leaching, defined as the “treatment of complex substances, such as a mineral, with a specific solvent, to separate its soluble parts from the insoluble ones” [2]. The heap leaching technology was pioneered in the U.S.A., but significant advancements were made to it in Chile [3], achieving industrial applications at large scale, perfecting and developing improvements in the methodology for obtaining minerals and applying them to different minerals, climates, and operations [4]. In addition to copper oxides, heap leaching is applied to a wide range of minerals, including copper sulphide minerals, such as chalcocite [5,6,7], covelline [8,9,10], or chalcopyrite [11,12,13]. On the other hand, non-metallic minerals, such as saltpeter, can also undergo leaching processes [14,15]. Applications of leaching in such cases have been studied by various authors [16,17,18] who have modeled mineral leaching at both laboratory and industrial scales.

Within the advance and generation of new technologies applied to the leaching of minerals is bioleaching, which involves the recovery of inorganic metals by means of microorganisms, using specific bacteria (or fungi) to extract a valuable mineral present in a mine or in a concentrate of a mineral and resulting in an acid solution containing metal in its soluble form. There are many types of bioleaching processes, and copper is the most common [19]. Bioleaching refers then to the process of extracting metals from ores using natural materials present in the environment, such as water, air, and microorganisms [20]. Essentially, bioleaching involves exploiting the capacity of specific bacteria and archaea, which occur naturally, to catalyze the oxidation of minerals for commercial purposes [21], or also for the treatment of mining waste or remediation, where microorganisms are used for the elimination of toxic metals and metalloids [22,23].

Bioleaching can be carried out using two methods: the direct method and the indirect method. In the direct method, easily oxidizable minerals are employed to initiate an enzymatic reaction with microorganisms that effectively separates the metal from the ore. On the other hand, in the indirect method of bioleaching, microorganisms do not come into direct contact with the minerals during the process, instead generating leaching agents that facilitate the mineral oxidation [24]. Among the main objectives of bioleaching are extracting metals from their ores in an efficient and economical manner, reducing the environmental impact of mining activities by using a natural and sustainable process, recovering metals from low-grade ores that would otherwise not be economically feasible to extract using traditional mineral processing methods, generating less waste compared to traditional mineral processing methods, obtaining high-purity metals suitable for various industrial applications, and reducing the consumption of energy and other resources required for metal extraction [20]. Bacterial leaching of metallic sulphides has developed quickly over the last few decades [25]. The utilization of microorganisms for the retrieval of heavy metals has become a well-established biotechnological practice [26,27], which has been used primarily in copper [25,28,29], cobalt [30], nickel [31], zinc [32], and uranium [33] recovery, metals that are typically obtained from sulfides that are insoluble or, in the case of uranium, from oxidized minerals [34].

Although bioleaching has become popular in industrial contexts, mainly associated with copper mining, there are also applications in relation to rare earth elements [35], such as: bioleaching of rare earths from monazite, using fungal strains such as *Aspergillus niger*, a strain of *Aspergillus terreus* and a *Paecilomyces* spp., where the least squares method of fitting is used to correlate the recovery of rare earths as a function of pH [36] and the relation between metabolites and concentrations of rare earth elements (REE) [37]; bioleaching of REE from phosphate rock using *Acidithiobacillus ferrooxidans*, obtaining better results than abiotic leaching [38,39]; extraction of Al and REE from red muds through two-stage aerobic and anaerobic bioleaching by *Acidianus manzaensis* with the addition of pyrite [40]; and bioleaching as a green recycling strategy, where the leaching of waste or recycled material (from electronic devices) is carried out by microorganisms (*Acidithiobacillus* and *Leptospirillum ferrooxidans*) [41,42]. From the above, it can be deduced that the research efforts in REE bioleaching have been focused on the feasibility study, not on the generation of phenomenological models that explain the dynamic of operation, which can be explained by the recentness of this field of research.

In the present work, an exhaustive analysis of the modeling techniques of theoretical representations of the bioleaching process is developed. The analysis is generally applied at the industrial scale to the leaching of sulphide minerals and is mainly developed through heap leaching. The structure of the work considers the definition of bioleaching processes in Section 2, exposing its dynamics and presenting a practical example (chalcopyrite bioleaching). Section 3 introduces the main microorganisms used in bioleaching (mainly at the industrial scale). In Section 4, the modeling of bioleaching dynamics is developed, a literature bibliometric analysis is developed, general schemes of conventional leaching modeling are introduced, a compilation of the different adjusted models in bioleaching processes is presented, and applications of machine learning techniques to the modeling of leaching dynamics with the use of microorganisms are shown. Finally, the conclusions and future perspectives are presented in Section 5.

## 2. Bioleaching Process

Bioleaching—as opposed to traditional (heap) leaching—has become an attractive alternative in recent decades due to its potential in mineral leaching on a laboratory scale and, mainly, due to its industrial applications [43]. The industrial operation applied to the leaching of copper sulfides considers the formation of a heap of material to be leached. Heap leaching requires ore preparation, primarily size reduction, to maximize mineral-leach interaction and placement of an impervious base to prevent leach loss and contamination of water bodies [44]. A drip irrigation system and sprinklers are installed on the heap to distribute a solution that is enriched with reagents and bacteria across the entire exposed area, microorganisms that feed on mineral elements (such as sulfur and iron) and that naturally separate the metal particles of interest from the impurities. In other words, the bacteria dissolve the rock, turning it into a solution which is collected at the base of the heap or slope by a system of pipes [45]. The dissolution of minerals is the product of the oxidation carried out by the bacteria of the inorganic compounds present in them, and this metabolic capacity of the bacteria is what allows a copper sulfate solution, from which the metal can be recovered in the classic stages of Solvent Extraction and Electrowinning [46].

**Figure 1:**
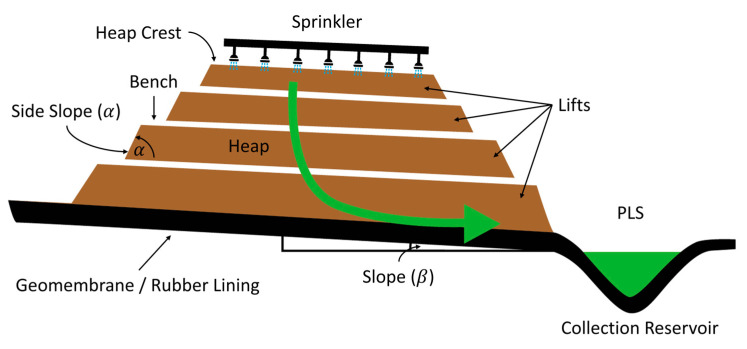
Bioleaching process in heaps on an industrial scale.

The variety of microorganisms used in bioleaching depends on various factors, such as the mineral to be leached or the operating conditions, such as pH or temperature [47]. The bioleaching process in heaps (see Figure 1) or in dumps includes a variety of physical, chemical, and biological sub-processes, among which are the study of solution flow, gas flow, heat flow, diffusion transport, microbial population dynamics, solution chemistry, ore leaching mechanisms, and granulometry [45].

Based on the literature review, different applications or case studies of microorganisms can be retrieved, such as chalcopyrite bioleaching (by using the microorganism *A. ferrooxidans*), either by a direct or indirect mechanism [19,48], or the bioleaching of pyrite and sphalerite [49]. In the indirect mechanism, bacteria oxidize soluble Fe^2+^ to Fe^3+^ and sulfur to sulphate and ferric ions oxidize the sulphide minerals in an acidic environment. The direct contact mechanism occurs when minerals that are easily receptive to oxidation create a direct enzymatic attack (adhering the bacteria to the mineral) using the microorganisms to separate the metal and mineral.

## 3. Microorganisms in Bioleaching

**Figure 2:**
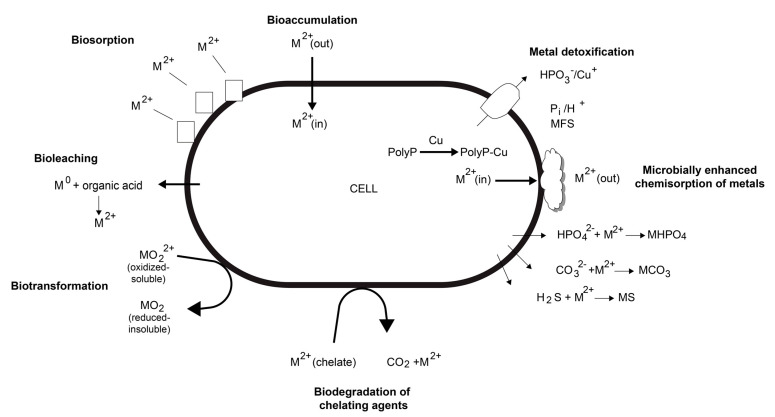
Scheme of typical interactions bacteria-metal. Modified from [50].

The interaction of microorganisms with metals occurs through many mechanisms, as shown in Figure 2. Metals are essential for the metabolism of all organisms, including microorganisms such as bacteria, which can be accumulated through the use of specific or general energy-dependent metal transporters. These transporters can directly incorporate the metals or utilize organic compounds, such as siderophores for iron, to chelate them. Certain transporters can also be employed to bioaccumulate metals within the cell, which is achieved through the sequestration of the metal by cysteine- or histidine-rich proteins or by chelating it with inorganic polyphosphates (polyP)—long chains of phosphate molecules linked through phosphodiester bonds that bear a highly negative charge at neutral pH. Biosorption, on the other hand, refers to the binding of metal ions throughout the biomass, while biomolecules present in the biomass have various chemical groups that act as ligands, facilitating the binding of metal ions.

Microorganisms are also capable of catalyzing different biotransformations, including the conversion of highly toxic metals into forms that are less volatile or less soluble; they also precipitate metals as insoluble carbonates, hydroxides, sulfides, and phosphates (constituting a biomineralization process) [50].

Bioleaching is a technique that dissolves metals in an aqueous medium through bacteria that release minerals in a greater quantity than with conventional methods, which can leach through direct action on the mineral or through the oxidation of an ion, which sometimes in turn works as an oxidizer of the target mineral. There are different types of bacteria, among which are those that can oxidize sulfur to sulfuric acid and arsenic to a species that is harmless to humans (which, along with reducing production costs, reduces sulfur indicators and arsenic released into the environment). This technology has a series of economic advantages due to low operating costs, while at the environmental level there is no emission of gases or dust, and the concentrates that contain high levels of metals (such as zinc, arsenic, sulfur, or other heavy metals derived from copper smelting processes) can be treated.

Bioleaching is an efficient and straightforward technology employed for the extraction of metals from low-grade ores and mineral concentrates. For instance, the recovery of metals from sulfide ores is typically facilitated through the activity of chemolithotrophic bacteria— *Thiobacillus ferrooxidans* and *T. thiooxidans* (the most commonly used types). These bacteria are capable of converting insoluble metal sulfides into soluble metal sulfates [20]. In the case of minerals that do not contain sulfides, these can be treated by heterotrophic batteries and by fungi.

Currently, the main application of bioleaching is for the retrieval of copper [28,29,51], uranium [52,53], and gold [54,55], while the principal techniques used are heap leaching, dump leaching, or leaching in situ [20]. The predominant microorganisms utilized in industrial-scale bioleaching operations are Gram-negative, non-spore-forming bacilli belonging to the *Thiobacillus* genus. These bacteria thrive in aerobic conditions and are typically chemolithoautotrophic, meaning they utilize atmospheric CO_2_ as a carbon source for the synthesis of new cell material. They derive energy from the oxidation of reduced or partially reduced sulfur compounds, including sulfides, elemental sulfur, and thiosulfate, with sulfate serving as the final product of oxidation. This type of leaching by bacterial microorganisms is carried out in an acid environment (1.5 < pH < 3), with the most common acidophilic species being *T. thiooxidans* and *T. ferrooxidans*. Species belonging to the same genus include *T. prosperus* and *T. cuprinos*, among others.

In addition, there exists the *Leptospirillum* genus, which includes the obligately chemolithotrophic and acidophilic ferrous iron-oxidizing bacterium, *Leptospirillum ferrooxidans*. This microorganism is capable of operating under lower pH conditions and higher concentrations of uranium, molybdenum, and silver. However, it is sensitive to copper and incapable of oxidizing sulfur or sulfur compounds.

Therefore, an important part of the bioleaching processes is based almost exclusively on the activity of *T. ferrooxidans*, *L. ferrooxidans* and *T. thiooxidans*, whose main functions are to convert highly insoluble metal sulphides through biochemical oxidation reactions into water soluble metallic sulphates. These metals can be liberated from minerals through either direct or indirect bacterial mechanisms. In direct bacterial leaching, the bacterial cell comes into direct contact with the surface of the sulfide mineral, and the oxidation process occurs through several enzymatic steps. On the other hand, in indirect bioleaching, the bacteria produce a leaching agent that chemically oxidizes the sulfide ore. In an acid solution, this leaching agent could be Fe^3+^ [24].

An example of direct bacterial leaching is the oxidation process of pyrite to iron (III) sulfate, according to the reactions below [24]:

$$4FeS_{2}+14O_{2}+4H_{2}O\overset{Bacteria}{\to }4FeSO_{4}+4H_{2}SO_{4}$$

$$4FeSO_{4}+O_{2}+4H_{2}SO_{4}\overset{Bacteria}{\to }2Fe_{2}{\left(SO_{4}\right)}_{3}+2H_{2}O$$

Best summarized by the following reaction:

$$4FeS_{2}+15O_{2}+2H_{2}O\overset{Bacteria}{\to }2Fe_{2}{\left(SO_{4}\right)}_{3}+2H_{2}SO_{4}$$

Or generically by:

$$MeS+2O_{2}\overset{Bacteria}{\to }MeSO_{4}$$

In contrast, for indirect bacterial leaching, the metal solubilization process can be represented by the reaction below (pH < 5):

$$MeS+Fe_{2}{\left(SO_{4}\right)}_{3}\to MeSO_{4}+2FeSO_{4}+S^{0}$$

The ferrous ion that arises from this reaction is reoxidized to ferric, and as such, it can participate again in the oxidation process. Bacterial oxidation of Fe^2+^ is approximately 10^5^–10^6^ times faster than the chemical oxidation. Elemental sulfur can be oxidized (by bacterial action) to H_2_SO_4_, as shown in the following reaction:

$$2S^{0}+3O_{2}+2H_{2}O\overset{Bacteria}{\to }2H_{2}SO_{4}$$

The efficiency of the bioleaching process is highly dependent on the effectiveness of the microorganisms used, as well as the chemical and mineralogical composition of the mineral being leached, so that the optimization of the metals depends directly on the bacteria’s optimal growth conditions. Among the main factors that influence bioleaching are nutrients, O_2_, CO_2_, pH, temperature, mineral substrate, heavy metals, and organic surfactants and extractants.

In industrial operations, bioleaching is started by adding sulfuric acid and aerating the heap, the temperature of which rises as leaching progresses. The first organisms to act are mesophilic acidophiles (temperature < 40 °C), which are mostly Gram-negative bacteria; next in succession are moderate thermoacidophiles (40–60 °C), which are mostly Gram-positive, and finally the extreme thermoacidophiles (>60 °C), which are mostly Archaea [56]. Microbial consortia dominated by autotrophic, acidophilic prokaryotes that oxidize iron or sulfur are commonly used in biomining processes. These consortia are typically utilized in stirred tank reactors and irrigated heaps. The growth environments within heap reactors are highly heterogeneous and change over time, resulting in a greater variety of microorganisms colonizing the heaps. For information on assembling microbial consortia for the processing of various minerals and concentrates, Rawlings and Johnson’s work [57] is a valuable resource to consult.

## 4. Modeling of Mineral Bioleaching

### 4.1. Process Modeling and Bibliometric Analysis

**Figure 3:**
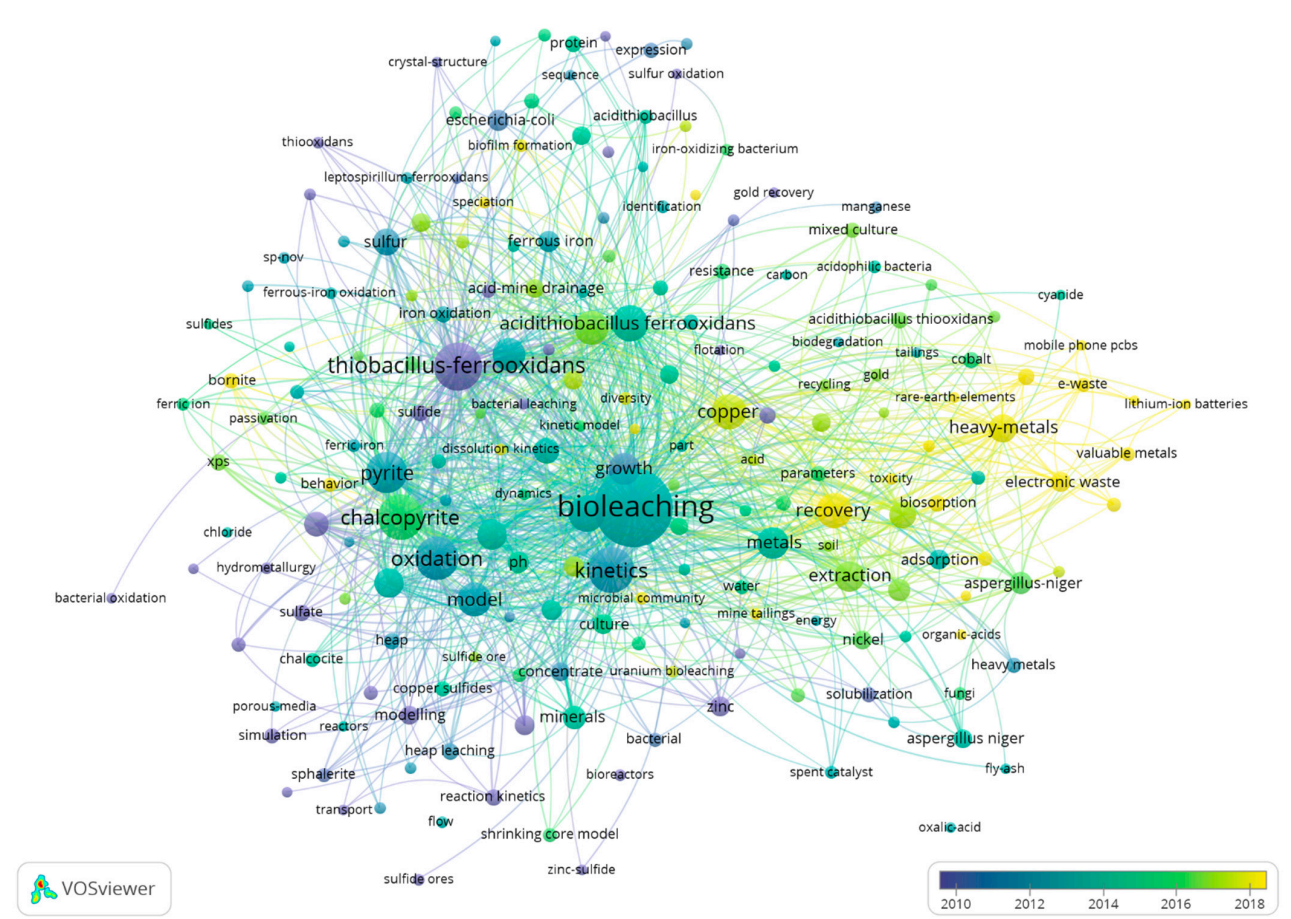
Visualization of networks of consulted bibliography (VOSviewer software, version 1.6.19).

Within the field of mineral processing, kinetic modeling approaches are important because they determine the reaction time needed or how fast or slow a reaction will be [47]. The bibliometric analysis (generated based on the references of the “Web of Science”) indicates that the modeling of the bioleaching process focuses on the study of dissolution, kinetics, oxidation, recovery, and adsorption, among others. The network in Figure 3 indicates the existence of different clusters in the modeling of the bioleaching process, relative to mineral bioleaching at an industrial scale (such as pyrite or chalcopyrite) and the dynamics of the functioning of the different microorganisms, although the focus in recent years has been the bioleaching of heavy metals and their recovery from electronic waste.

According to a search conducted on the Web of Science in March 2023, a total of 2067 scientific articles have been published on mineral bioleaching that include modeling in their approach. The number of articles published on the bioleaching of minerals with modeling has increased significantly in recent decades, suggesting a growing interest in this area of research. Finally, some of the most frequent topics in articles on bioleaching of minerals with modeling include kinetic modeling of bacterial leaching, simulation of leaching processes, and optimization of leaching parameters.

### 4.2. Leach Modeling

Bioleaching has emerged as a predominant technological route in mineral recovery, mainly in low-grade mineral processing at an industrial scale. The development of bioleaching technologies has emerged as an alternative to conventional leaching methods, and in its beginnings, the formulation of theoretical or phenomenological models revealed that the processes underlying even heap leaching of oxidized minerals are physically complex. The adoption of bioleaching has heightened the intricacy of the procedure, and consequently of the related models.

During bioleaching, oxidants react and extract metal. These oxidations processes, just like in conventional leaching, is controlled by any of the following processes, or mixtures of them [58]:

1. Diffusion-controlled process through the product layer: The creation of a layer of product around the material can impede the diffusion of the oxidant to the material’s surface, resulting in a deceleration of the leaching process.
2. Chemically controlled process: The product layer is absent or its presence does not affect the free movement of the oxidant. The reaction between the surface and the reactant is slower than the diffusion of the oxidant.
3. Film diffusion process: The oxidant movement to the surface can be hindered by the bulk leach solution, resulting in slower leaching kinetics.

When there is no product layer on the surface of the material, the material size decreases gradually over time as a result of solubilization, also known as particle shrinkage. When the product layer hinders the movement of the oxidant, leaching is reduced, and this is referred to as diffusion-controlled leaching dynamics. On the other hand, when the product layer is non-resistive or absent, and the particle shrinks, the process is chemically controlled [58].

Expressions for the conversion of particles according to the SCM model are shown in Table 1, where α is the leached metallic fraction, e is the thickness, L is the length, $r_{n}$ and $r_{ext}$ are the radius of the nucleus and the particle, $C_{B}$ is the molar density of the metal in the mineral, a is the stoichiometric coefficient of the fluid, b is the stoichiometric coefficient of the solid, $D_{AB}$ represent the effective diffusivity coefficient, τ is the complete conversion time, k is the kinetic coefficient, and $C_{A0}$ is the initial concentration of the fluid. On the other hand, the equations in Table 1 consider that the oxidant concentration is constant, which is not usually the case at a practical level, so conventional kinetic models are modified to consider variations in the concentration of leaching agents, such as is presented in Equation (1).

(1) $$\frac{t}{\tau }=k_{a}^{\prime }\int_{0}^{t}Cdt$$

Finally, it should be considered that in an important part of the cases, it is not possible to assign a single control stage to a transformation, so it is necessary to adopt the possibility that the control is of a mixed type, that is, that two stages govern the transformation. Additionally, Arrhenius developed a temperature-dependent reaction rate equation (see Equation (2)) showing that the kinetic constant k=1/τ increases exponentially with temperature as shown in Equation (2) [59], where T is the temperature in absolute terms, R, the universal gas constant, and $E_{a}$, the activation energy.

(2) $$k=k_{0}e^{-\frac{Ea}{RT}}$$

### 4.3. Bioleaching Modeling

Bioleaching modeling has not been far from controversy, which has to do with both the bioleaching mechanism (direct or indirect) and the kinetic modeling. The direct mechanism is based on the fact that sulphides act as nutrients for the bacteria, and it is assumed that the growth dynamics of the microorganism are in accordance with the Monod equation [60]; however, some of them assume that the character of the bacteria on the surface and in solution must agree with Langmuir’s isothermal equation [61]. According to the direct mechanism, the bacteria is adsorbed on the surface of sulfide minerals and oxidize sulfides. On the other hand, under the indirect mechanism, bacteria oxidize Fe^2+^ to Fe^3+^, and then Fe^3+^ oxidizes sulfides [62].

Among the first models developed to represent the bioleaching of minerals are the bacterial mass balances, which indicate the variation in the number of bacteria attached to a particle, which can be expressed as shown in Equation (3), while the variation of bacteria in the solution is shown in Equation (4) [63].

(3) $$\frac{dN_{A}}{dt}=\frac{\mu_{m}S}{K_{S}+S}+K_{A}N_{F}\left(N_{S}-N_{A}\right)-K_{D}N_{A}$$

(4) $$\left(1-F\right)\frac{dN_{F}}{dt}=K_{D}N_{A}n-K_{A}N_{F}\left(N_{S}-N_{A}\right)n$$

where $N_{A}$ is the number of cells adsorbed on a single carbon particle; S is the concentration of pyritic sulfur in a single coal particle; $N_{s}$ is the cells required to saturate the particle surface; $N_{F}$ is the concentration in number of free cells in the liquid phase; $K_{A}$ and $K_{D}$ are cell adsorption and desorption coefficients; and $\mu_{m}$ and $K_{S}$ are the maximum specific growth rate of the cells and the saturation constant, respectively. The first term of Equation (3) is the rate of increase in cell number as a result of pyrite oxidation; the second and third terms are the cell adsorption and desorption rates, respectively. Additionally, F is the volume fraction of the coal–water mixture that is occupied by coal particles and n is the coal particles per unit volume of the coal water mixture [63].

Herrera et al. [60] developed a model to account for the bioleaching kinetics of complex sulphide minerals; the model considers the indirect mechanism, where the bacteria oxidize the ferrous ion to ferric, which in turn acts as an oxidizing agent. Considering the ferrous ion as the only limiting substrate, the Monod growth rate for both absorbed and free bacteria is presented in Equation (5). The number of bacteria absorbed on the surface of the mineral and in free solution were established as shown in Equations (3) and (4) [63], while according to the SCM model proposed by Braun et al. [64], in addition to that developed by Madsen et al. [65], the rate of decrease in the radius of the unreacted nucleus $\left(r_{c}\right)$ of a given mineral species is given by Equation (6).

(5) $$\mu =\mu_{max}\frac{Fe^{2+}}{K_{Fe^{2+}}+Fe^{2+}}$$

(6) $$-\frac{dr_{C}}{dt}=\frac{M_{S}}{\rho G\phi }\frac{\left[Fe^{3+}\right]}{\left[\frac{1}{G\beta }+\left(\frac{\sigma }{D_{eff}}\right)\left(\frac{r_{C}}{R}\right)\left(R-r_{C}\right)+\left(\frac{1}{K_{C}}\right){\left(\frac{r_{C}}{R}\right)}^{2}\right]}$$

Bhattacharya et al. [66], on the other hand, proposed a reaction kinetic model for chalcopyrite bioleaching using *Thiobacillus ferrooxidans*; the model incorporates the reduction of particle size during the leaching process, and the rate for the most important components are described as shown in Equations (7)–(9), where $C_{A}$, $C_{X},$ and $C_{R}$ are the CuFeS_2_, cells, and CuSO_4_ concentrations, respectively. μ is the specific growth rate, and $K_{A}$, $Y_{A}$, and k are constant. In addition, it works under the following assumptions: the mass of the bacteria produced per the converted substrate is constant $\left(\frac{dC_{X}}{dt}=-Y_{A}\frac{dC_{A}}{dt}\right)$, the particle size decreases continuously following the SCM model, and the process is not controlled by diffusion in film layer.

(7) $$\frac{dC_{A}}{dt}=-\frac{\mu_{max}C_{A}}{K_{A}+C_{A}}\frac{C_{X}}{Y_{A}}$$

(8) $$\frac{dC_{X}}{dt}=\frac{\mu_{max}C_{A}}{K_{A}+C_{A}}C_{X}$$

(9) $$\frac{dC_{R}}{dt}=k\frac{\mu_{max}C_{A}}{K_{A}+C_{A}}\frac{C_{X}}{Y_{A}}$$

Another work that validates the SCM model in the context of finely ground particle leaching columns is the work developed by Neuburg et al. [67], which captures the effects that occur both at the particle and cluster scales in the context of bacterial leaching. Neuburg et al. [67] represent the mass balance for the chemical species in the system by means of a continuity equation in the axial direction (see Equation (10)); the variation in the quantity of adhered bacteria along the column is expressed as is shown in Equation (11), and the variation of bacteria in solution is shown in Equation (12).

(10) $$\frac{\partial \left(\epsilon_{s}C_{i}\right)}{dt}+\upsilon_{z}\frac{\partial C_{i}}{dz}-\frac{\partial }{dz}\left(D_{ai}\frac{\partial C_{i}}{dz}\right)=\sum R_{i}$$

(11) $$\nu_{Z}\epsilon_{S}\left(1-\epsilon_{L}\right)\frac{dX^{\prime }}{dz}=\mu X^{\prime }\left(1-\epsilon_{L}\right)+K_{ads}X\left(X_{sat}-X^{\prime }\right)\left(1-\epsilon_{L}\right)-K_{des}X^{\prime }\left(1-\epsilon_{L}\right)$$

(12) $$\nu_{Z}\frac{dX}{dz}=\epsilon_{S}X\mu -K_{ads}X\left(X_{sat}-X^{\prime }\right)\left(1-\epsilon_{L}\right)+K_{des}X^{\prime }\left(1-\epsilon_{L}\right)$$

where $\epsilon_{L}$ represents the empty fraction of the deposit, X′ is the concentration of cells attached per unit volume, X the concentration of bacteria, $K_{ads}$ and $K_{des}$ are the absorption and desorption constants, and $X_{sat}$ is the saturation concentration of bacteria. Equation (13) show the total quantity of bacteria per unit volume of solution at any point in the column participating in the oxidative process, while the oxidation rate of Fe^2+^ is defined as shown in Equation (14).

(13) $$X_{T}=X+\frac{\left(1-\epsilon_{L}\right)}{\epsilon_{S}}X^{\prime }$$

(14) $$-R_{Fe^{2+}}=\mu_{max}\frac{\epsilon_{S}}{Y}X+X^{\prime }\frac{\left(1-\epsilon_{L}\right)}{\epsilon_{S}}\frac{\left(Fe_{L}^{2+}\right)}{\left(K+\left(Fe_{L}^{2+}\right)\right)}F_{1}\cdot F_{2}$$

where $F_{1}$ depends on $\mu_{max}$ and the pH of the solution, and $F_{2}$ depends on $\mu_{max}$ and the concentration of dissolved oxygen, being a term that becomes less than one when the level of dissolved oxygen in the solution limits speed. K, on the other hand, represents the bacterial saturation constant [67]. Then, the leaching rate of sulfides contained in the mineral particle anywhere in the column is modeled as shown in Equation (15), while that leaching rate of the sulfides contained in a mineral particle anywhere in the column can be established as shown in Equation (16), where $r_{io}$ and $r_{i}$ are the radius of the particle and the radius of the reaction front, respectively, $\phi_{io}$ the shape factor of the particle, G the general grade of the mineral, β the true oxidation kinetic constant of the mineral, σ the stoichiometric factor, and the mass transfer coefficient of Fe^3+^ in the liquid film is denoted by $k_{c}$. Then, the reacted fraction of the mineral particles can be defined according to the processes that control the reaction (see Table 1).

(15) $$X_{T}=X+\frac{\left(1-\epsilon_{L}\right)}{\epsilon_{S}}X^{\prime }$$

(16) $$-R_{CuFeS_{2}}=\frac{4\pi r_{i}^{2}}{\phi_{io}}\frac{\left(Fe_{L}^{3+}\right)}{\left[\frac{1}{G\beta }+\frac{\sigma }{D_{eff}}\frac{r_{i}}{r_{io}}\left(r_{io}-r_{i}\right)+\frac{\sigma }{K_{c}}{\left(\frac{r_{i}}{r_{io}}\right)}^{2}\right]}$$

Asai et al. [61] studied the kinetics of bacterial dissolution of FeS_2_ particles by *T. ferrooxidans*, carrying out experiments on the adsorption of bacteria on the surface of FeS_2_ and the bacterial dissolution of this mineral. The adsorption equilibrium is modeled using the Langmuir equation (see Equation (17)), where $X_{A}$ is the concentration of absorbed bacteria, $X_{L}$ is the free bacteria in liquid phase, $K_{A}$ is the adsorption equilibrium constant, and $X_{Am}$ is the capacity maximum adsorption. The kinetic model developed to describe the behavior of pyrite dissolution caused by direct microbial action was modeled using the total growth rate of bacteria, as shown in Equation (18) [68].

(17) $$X_{A}=\frac{K_{A}X_{Am}X_{L}}{\left(1+K_{A}X_{L}\right)}$$

(18) $$\frac{dX_{T}}{dt}=R_{A}+R_{L}\;X_{T}=X_{A}\left(\frac{W_{0}}{V}\right){\left(1-\alpha \right)}^{\frac{2}{3}}+\left(1-\phi \right)X_{L}\;R_{A}=\mu_{A}X_{A}\left(\frac{X_{Am}-X_{A}}{X_{Am}}\right)\left(\frac{W_{0}}{V}\right){\left(1-\alpha \right)}^{\frac{2}{3}};R_{L}=\mu_{L}X_{L}\left(1-\phi \right)$$

Casas et al. [69] developed a model (see Equation (19), oxidation rate based on the Michaelis–Menten equation that considers oxygen as a limiting substrate) for the bioleaching of copper sulfide minerals (chalcocite and/or pyrite) relating the dissolution rate of ore with the rate of oxidation by bacteria attached to the mineral surface, where FPY is the rate of the mass of pyrite and chalcocite leached.

(19) $$\frac{d\alpha }{dt}=\frac{M_{Ch}M_{Py}}{5/2M_{Ox}M_{Py}+7/2\left(FPY\right)M_{Ox}M_{Ch}}\frac{1}{\rho_{B}G^{o}}XV_{m}\left(\frac{C_{L}}{K_{m}+C_{L}}\right)$$

Mehta et al. [70] indicate that the biodissolution of metals follows a SCM model, where the leaching is controlled by diffusion of the leaching agent through the product layer (see Table 1) [71]. Sidborn et al. [72], on the other hand, developed a model for representing the process of bioleaching of secondary copper ores from a heap leach. It was considered that the aeration of the heap is explained by natural convection caused by the fluid pressure gradient. The transport of Fe^3+^ from the surface to the reaction zone is computed considering the diffusion of the film, the diffusion within the particle, and the reaction kinetics (see mass balance in Equation (20)), and the rate of decrease of the radius of the unreacted nucleus for a given mineral species is presented in Equation (16) [65], where $D_{L}$ is the dispersion, $\varepsilon_{L}$ is the liquid volume fraction, $q_{L}$ is the flow rate, and $R_{i}$ is the reaction rate of certain specie i.

(20) $$\varepsilon_{L}\frac{\partial C_{i}}{\partial t}=D_{L}\varepsilon_{L}\nabla^{2}C_{i}-q_{L}\nabla C_{i}+R_{i}$$

Then, Wang et al. [62] modeled the bioleaching of chalcopyrite catalyzed by silver ions Ag^+^ (as a leaching agent), establishing a kinetic model based on the SCM model presented in Equation (6) [64,65], where the Fe^3+^ concentration in the radius change model of the mineral particle is replaced by the Ag^+^ concentration, and the inclusion of the leaching rate $\alpha =1-{\left(r_{i}/r_{i0}\right)}^{3}$ results in a mathematical model for the silver ion-catalyzed bioleaching of chalcopyrite. The copper recovery obtained from Wang et al. [62] in experimental tests agrees with that calculated from the kinetic model.

Leahy et al. [73] modeled liquid flow, bacterial transport, and depletion of copper sulfide using the SCM (surface reaction and diffusion controlled) model, capturing effects that occur at both particle and cluster scales. The model also incorporates a stack heat flow model (see Equation (22)), which is dependent on bacterial temperature. The SCM model is then coupled to equations involving reactions catalyzed by bacteria (transport equation for the concentration of species in the liquid in Equation (20)). The model to represent the extractions formulated by Leahy et al. [73] is given by the SCM model, which is similar to those previously formulated by Neuburg et al. [67], as shown in Equation (21), where $\alpha_{i}$ represents the mineral recovery fraction, $\sigma_{i}$ is the stoichiometric coefficient, φ is the shape factor of the particle, $\beta_{i}$ is the intrinsic oxidation rate, $D_{eff}$ is the effective diffusion coefficient, and $k_{A,i}$ is the ith Arrhenius rate function [74].

(21) dαidt=31−αi23CFe3+τc,i+6τd1−αi131−1−αi13τc,i=δφρbMFekA,iβiMore,i;τd=δ2σiGiφρbMFeDeffMore,i

(22) $$\sum_{i=l,g,r}\varepsilon_{i}\rho_{i}C_{p,i}\frac{\partial T}{\partial t}=k_{B}\nabla^{2}T-\varepsilon_{g}\rho_{g}C_{p,g}\nu_{g}\frac{\partial T}{\partial y}+\varepsilon_{L}\rho_{L}C_{p,L}\nu_{L}\frac{\partial T}{\partial y}+Q$$

Then, Leahy et al. [75] indicate that the difference in the flow rate of liquid has a significant effect on the copper extraction, while in the comparison between moderate thermophiles (MT) and mesophiles in heap bioleaching, the leaching kinetics is higher in the heap upper part due to the capability of the leaching agent to cool the heap and allow microorganisms to initially survive only in the top region.

Lizama et al. [76] studied the bioleaching kinetics of ore containing sphalerite and pyrite, which shows biphasic behavior and is composed initially for a colonization phase, followed by a steady state phase, which is described in Equation (23), where k′ is the observed constant (described by a logistic equation) [77], μ is the bacterial growth rate constant (which is described by Monod’s equation), and the true value of the rate constant is given by k. Bacterial cells attached to sphalerite preferentially over pyrite, which stimulated bacterial reproduction more than sphalerite, although sphalerite could harbor a larger number of cells. At a steady state, k and $k_{0}^{\prime }$ for ZnS were much faster than those for FeS_2_. Subsequently, Lizama et al. [78] fitted a model to represent bioleaching kinetics at various heights (both in heaps and columns) of zinc sulfide. Bioleaching kinetics of sphalerite and pyrite matched the SCM model of colonization [76]. It should be noted that in the study by Lizama et al. [78] columns irrigated at the same rate showed μ that increased linearly with inverse column height, whereas bioleaching kinetics in the heaps were proportional to irrigation rate over column height (L/h), while common values k,μ, and $k_{0}^{\prime }$ are functions of irrigation rate (L) and column height (h).

(23) 1−31−α23+21−α=k′t;k′=k0′eμt1−k0′k1−eμt

(24) $$k,\mu ,k_{0}^{\prime }=f\left(L/h\right)$$

Petersen et al. [32] describe a modeling study of the bioleaching process in heaps to model the overall rate of Zn extraction as a function of gas–liquid oxygen mass transfer, factors that affect acid delivery to the heap, and factors that affect the temperature distribution inside the heap. Diffusion’s transport is the main mode of transport of dissolved components to and from the moving solution and through cracks and/or fissures between particles and pore spaces of the particles. The mathematical formulation, solved by means of the reaction diffusion equation and written to describe the transport through a spherical particle, is given by Equation (25), while the transport of a solute i in the vertical direction axial z is then represented by Equation (26). Additionally, Petersen et al. [45] describe the mathematical modeling of mineral kinetics (see Equation (27)), that is, the mineral conversion rate and microbial kinetics (see Equation (28)), in addition to the combined mathematical modeling of diffusion advection, among other models that are summarized in Petersen [79].

(25) $$\frac{\partial c_{i}}{\partial t}=\frac{D}{\tau^{2}}\left(\frac{\partial^{2}c_{i}}{\partial r^{2}}+\frac{2}{r}\frac{\partial c_{i}}{\partial r}\right)-\sum_{j}\frac{S_{i,j}}{\varepsilon }$$

(26) $$\frac{\partial C_{i}{\left.\right|}_{r=R}}{\partial t}+\frac{G_{L}}{\varepsilon_{f}\rho_{h}}\frac{\partial C_{i}{\left.\right|}_{r=R}}{\partial z}=-3\frac{D_{i}}{R\tau^{2}}\frac{\varepsilon_{s}}{\varepsilon_{f}}\frac{\partial C_{i}}{\partial r}{\left.\right|}_{r=R}$$

(27) $$\frac{dX}{dt}=k(T,d_{0})f(C){\left(1-X\right)}^{\varphi }$$

(28) $$\frac{dc_{x}}{dt}=c_{x}k_{g}\left\{f_{g}\left(T\right)\left[\Pi \left(k_{e}+1\right)-k_{e}\right]-k_{d}f_{d}\left(T\right)\right\}$$

Vilcáez et al. [80] formulated a model to study the autothermal performance of a heap that uses a mixture of mesophilic (M) and thermophilic (T) microbes for Cu recovery from CuFeS_2_, finding that the maximum performance occurs under minimum biomass irrigation concentrations and maximum leach solution flow rates. The model formulated to represent ore dissolution from copper sulfides is a function of variables such as copper grade, the density of sulfide minerals, the fraction of liquid in the heap, and respiration rates of mesophiles and thermophiles in the fluid, as shown in Equation (29), where α is the copper recovery fraction, β is a proportionality factor, $G_{min}$ is the grade of copper, $r_{O_{2},M}$ and $r_{O_{2},T}$ are the respiration rates in the fluid mass, $r_{O_{2},M}^{\prime }$ and $r_{O_{2},T}^{\prime }$ are the respiration rates of at the surface, $q_{i}$ is the maximum specific respiration rate, $K_{m}$ is Monod’s mean growth rate constant, $C_{L}$ is available oxygen, and $X_{i}$ is the concentration in the fluid volume.

(29) $$\frac{d\alpha }{dt}=\frac{\beta }{\rho_{min}G_{min}}\left(\theta r_{O_{2},M}+r_{O_{2},M}^{\prime }+\theta r_{O_{2},T}+r_{O_{2},T}^{\prime }\right)\;r_{O_{2},i}=q_{i}\frac{C_{L}}{K_{m}+C_{L}}X_{i};r_{O_{2},i}^{\prime }=q_{i}\frac{C_{L}}{K_{m}+C_{L}}X_{i}^{\prime }|\forall i\in \left\{M,T\right\}$$

Continuing with bioleaching modeling, Bouffard and Dixon [81] modeled bioleaching in columns (using the HeapSim model [82], a numerical simulation tool developed to simulate the bioleaching process of minerals in heaps or heaps, allowing the evaluation of different operating and control scenarios and optimizing the process of extracting valuable metals from low-grade ores), focusing their efforts on finding the optimal conditions based on variables such as the biological parameters of the iron and sulfur oxidizing microorganims. The analysis showed that the crucial factor impeding the transition from particle kinetics to gas–liquid oxygen mass transfer was the rate-limiting step, which was found to increase with rising temperature, a greater proportion of fine pyrite grains, and higher grades of pyrite head. Additionally, the oxidation of pyrite was hindered by the competition for oxygen between sulfur- and iron-oxidizing microorganisms, which decreased the potentials and slowed down the process. The oxidation rate of pyrite is given by Equation (30) and the biological oxidation rate of Fe^2+^ is given by Equation (31), where Y is the cell concentration, $K_{Fe^{2+}}$ and $K_{O_{2}}$ are the saturation constants of ferrous and oxygen, $k_{Fe}$ is the maximum growth rate for mesophiles, moderate thermophiles, or extreme thermophiles, and $f_{g}\left(T\right)$ is a function of temperature. Additionally, the transport model is inspired by the aggregate model of the study of the hydrodynamics of conventional heap leaching [83].

(30) $$r_{FeS_{2}}=G_{FeS_{2}}\frac{dX}{dt}=G_{FeS_{2}}k_{0}e^{\left[-\frac{E_{a}}{R}\left(\frac{1}{T}-\frac{1}{T_{0}}\right)\right]}\sqrt{\frac{C_{Fe^{3+}}}{C_{Fe^{2+}}}}{\left(1-X\right)}^{\varphi }$$

(31) $$r_{Fe^{2+}}=\frac{dC_{Fe^{2+}}}{dt}=-\sum_{k}\frac{Y_{Fe,k}^{tot}f_{g}\left(T\right)k_{Fe}}{y_{Fe}}\left(\frac{C_{Fe^{2+}}}{K_{Fe^{2+}}+C_{Fe^{2+}}}\right)\left(\frac{C_{O_{2}}}{K_{O_{2}}+C_{O_{2}}}\right)$$

In Yin et al. [84], an integral model of the heap bioleaching process was developed to investigate the interaction dynamics between chemical reactions (mass balance according to Fick’s diffusion law) [85], the solution flow [86], the air flow described by the NS equations [69], the transport of solutes within the leaching system [87], and the energy balance [88]. There are two ways in which the oxidation rate is impacted by the flow of liquid: firstly, the movement of water helps to distribute heat and diminishes the temperature difference, and secondly, the cooling influence of the liquid flow can prevent the cessation of the bacterial-driven oxidation process by curbing the temperature increase in specific areas of the heap. The Michaelis–Menten equation characterizes the proportion of copper extracted through dissolved oxygen and bacteria (see Equation (19)).

In Ahmadi et al. [89] (in the presence of iron and sulfur oxidizing microorganisms), the dynamics of a bioleaching process was described with a kinetic model based on combined reactions (see leaching kinetics $r_{j}$ in Equation (32)). X is the mineral conversion rate, K(T) is a rate constant (function of temperature and initial granulometry), f(C) represents the solution composition (such as concentrations of Fe^3+^, Fe^2+^, protons, etc.), and g(X) is the fraction of unreacted mineral, representing the variation in topology of surface of a mineral grain throughout the leaching process. The modeling of the biological oxidations of Fe^2+^ and S^0^ are presented as forms of Monod’s expression, which are shown in Equations (33) and (34), respectively. The model proposed by Ahmadi et al. [89] is another application of how microorganisms are used as an indirect bioleaching mechanism.

(32) $$r_{j}=\frac{dX_{j}}{dt}=K_{j}\left(T\right)f_{j}\left(C\right)g_{j}\left(d_{0},X_{j}\right)$$

(33) $$r_{Bac,Fe}=Y_{Fe}f_{g,Fe}\left(T\right)\frac{\left[O_{2}\right]}{K_{O,Fe}+\left[O_{2}\right]}\cdot \frac{\left[Fe^{2+}\right]}{K_{Fe^{2+}}+\left[Fe^{2+}\right]}\cdot \frac{K_{Y,Fe}}{K_{Y,Fe}+Y_{Fe}}\;\cdot \left[1-e^{\left(-\frac{\left[H_{2}SO_{4}\right]}{K_{H,Fe}}\right)}\right]\left(\frac{k_{g,Fe}}{y_{g,Fe}}+k_{m,Fe}\right)$$

(34) $$r_{Bac,S}=-\frac{dg_{S}}{dt}=2Y_{S}\cdot f_{g,S}\left(T\right)\cdot \frac{\left[O_{2}\right]}{K_{O,S}+\left[O_{2}\right]}\cdot \frac{g_{S}}{K_{S}+g_{S}}\cdot \frac{K_{Y,S}}{K_{Y,S}+Y_{S}}\cdot \left(\frac{k_{g,S}}{y_{g,S}}+k_{m,S}\right)$$

On the other hand, studying the dynamics of uranium leaching considering the combination of solute transport equation models with microbial chemical reactions, Zhang et al. [90] developed an inverse system in order to identify the parameters of the proposed mathematical model. The one-dimensional solute transport model used by Zhang et al. [90] was established as the convection–diffusion equation [91] and the combination of chemical reactions with the convection–diffusion equations are presented in Equation (35), which are obtained using the model Schlogt’s molecular chemical kinetics [92,93], where $c_{1}$, $c_{2}$, and $c_{3}$ are the concentrations of hexavalent uranium ions, Fe^2+^, and Fe^3+^ in the liquid phase, respectively; and $s_{1}$ and $s_{2}$ are the concentrations of $FeS_{2}$ and $UO_{2}$ in the feed, respectively.

(35) $$\frac{\partial c\left(x,t\right)}{\partial t}=D\frac{\partial^{2}c\left(x,t\right)}{\partial x^{2}}-\nu \frac{\partial c\left(x,t\right)}{\partial x}\;\left\{\begin{array}{c} \frac{\partial c_{1}}{\partial t}=\frac{\partial^{2}c_{1}}{\partial x^{2}}-\nu \frac{\partial c_{1}}{\partial x}+k_{2}s_{2}^{2}+k_{5}s_{2}c_{3}^{2},\\ \frac{\partial c_{2}}{\partial t}=\frac{\partial^{2}c_{2}}{\partial x^{2}}-\nu \frac{\partial c_{2}}{\partial x}+k_{3}s_{1}c_{3}^{14}+k_{5}s_{2}c_{3}^{2}-k_{4}c_{2}^{4},\\ \frac{\partial c_{3}}{\partial t}=\frac{\partial^{2}c_{3}}{\partial x^{2}}-\nu \frac{\partial c_{3}}{\partial x}+k_{1}s_{1}^{4}-k_{3}s_{1}c_{3}^{14}+k_{4}c_{2}^{4}-k_{5}s_{2}c_{3}^{2}, \end{array}\right.$$

Then, Yaghobi et al. [94] generated a (comparative) analysis of mathematical models (one-dimensional) for simulating the (bio)leaching process. For this purpose, they used: a diffusion model, whose diffusion mechanism in the gas or aqueous phase can be considered as dimensionless and approximated by the law of Fick, as shown in Equation (36) [95]; a diffusion–advection model, where it is considered that the aqueous components are transported through the pores through advection, dispersion, and molecular diffusion processes [91,96] (see Equation (37)); a diffusion–reaction model (see Equation (38)), which is a special case of the advection, reaction, and diffusion equation; and a diffusion–advection–reaction model, shown in Equation (39) [97]. The methods applied by Yaghobi et al. [94] to provide solutions to Equations (36)–(39) include the homotopic perturbation method (HPM), the finite volume method, and the analytical method (Laplace).

(36) $$\frac{\partial C}{\partial t}=D_{x}\frac{\partial^{2}C}{\partial x^{2}}$$

(37) $$\frac{\partial C}{\partial t}=D_{x}\frac{\partial^{2}C}{\partial x^{2}}-v_{x}\frac{\partial C}{\partial x}$$

(38) $$\frac{\partial C}{\partial t}=D_{x}\frac{\partial^{2}C}{\partial x^{2}}-KC$$

(39) $$\frac{\partial C}{\partial t}=D_{x}\frac{\partial^{2}C}{\partial x^{2}}-\upsilon_{x}\frac{\partial C}{\partial x}-KC$$

On the other hand, in Govender-Opitz et al. [98] a hydrodynamic model is presented that describes the kinetics of leaching and the microbial dynamics within heap bioleaching. This study examines the distribution of microorganisms between the bulk-flowing pregnant leach solution (PLS) and the ore-associated phases within the ore bed. It considers how microbial transport occurs between these phases, hypothesizing that the movement of microorganisms between the bulk-flowing PLS and the mineral-associated phases is influenced by the concentration gradient of microorganisms between the two phases. Advection and dispersion forces assist in microbial colonization and transport through the mineral bed. The mineral’s inherent dissolution rate was calculated by dividing the total concentration of ferric iron by the total concentration of ferrous iron (as described in Equation (40)), while to better approximate the mineral leaching rate ($r^{R}$, see Equation (41)), a population balance model (PBM) was integrated into the hydrodynamic model. This allowed for the estimation of mineral dissolution rates that accounted for the available surface area, thus avoiding assumptions of particle size and shape homogeneity. $k_{m}$ is a constant rate, $A^{p}$ is the surface area of the particle, $l_{o}$ is the initial size distribution, $M^{p}$ is the particle mass, $V^{R}$ is the reactor working volume, $\phi_{MS}$ the CuFeS_2_ fraction, $I\left(\theta \right)$ the residence time distribution, and $N^{T}$ is the estimated total number of particles.

(40) $$r_{mineral}^{\prime \prime }=k_{m}{\left(\frac{C_{{Fe}^{3+}}}{C_{{Fe}^{2+}}}\right)}^{n}$$

(41) $$r^{R}=\int_{0}^{\infty }\int_{0}^{\infty }r_{mineral}^{\prime \prime }A^{p}\left(\theta ,l_{0}\right)\frac{M^{p}\left(\theta ,l_{0}\right)}{V^{R}}\phi_{MS}N^{T}f_{o}\left(l_{o}\right)I\left(\theta \right)d\theta dl_{0}$$

Additionally, Govender-Opitz et al. [98] modeled the microbial concentration in the total volume of the reactor $C_{x,total}$, as shown in Equation (42), where the advection–dispersion phenomenon that incorporates microbial growth is modeled to predict both temporal and spatial changes in microbial concentration. The microbial concentration in the flowing mass (PLS) $C_{x,PLS}$, and the phases associated with the mineral $C_{x,ore}$, is given by Equations (43) and (44), respectively [99].

(42) $$\frac{\partial C_{x,total}}{\partial t}=\mu_{x,total}\cdot C_{x,total}+D_{Z}\cdot \frac{\partial^{2}C_{x,total}}{\partial z^{2}}-v\frac{\partial C_{x,total}}{\partial z}$$

(43) $$\frac{\partial C_{x,PLS}}{\partial t}=\mu_{x,PLS}\cdot C_{x,PLS}+D_{Z}\cdot \frac{\partial^{2}C_{x,PLS}}{\partial z^{2}}-v\frac{\partial C_{x,PLS}}{\partial z}-k_{att}\left(C_{x,PLS}-C_{x,ore}\right)\;+k_{det}\left(C_{x,ore}-C_{x,PLS}\right)$$

(44) $$\frac{\partial C_{x,ore}}{\partial t}=\mu_{x,ore}\cdot C_{x,ore}+k_{att}\left(C_{x,PLS}-C_{x,ore}\right)-k_{det}\left(C_{x,ore}-C_{x,PLS}\right)$$

Noei et al. [100] modeled the kinetics of copper bioleaching from low-grade ores by microbial leaching, mainly studying the effect on bioleaching of pulp density and nutrient media. The effect of the density of the pulp on the bioleaching kinetics was examined using Da Silva’s method [101] and restricted multiple linear regression analysis (for the estimation of the term τ of Equation (45)) [102], while the kinetics of dissolution followed the SCM model. The method developed by Da Silva [101] introduces a delay, which is based on the mixed control mechanism shown in Equation (46).

(45) $$\tau =\tau_{F}X+\tau_{P}\left[1-3{\left(1-X\right)}^{\frac{2}{3}}+2\left(1-X\right)\right]+\tau_{R}\left[1-{\left(1-X\right)}^{\frac{1}{3}}\right]\;\tau_{F}=\frac{\rho_{S}R_{0}}{3K_{1}C_{ab}};\tau_{P}=\frac{\rho_{S}R_{0}^{2}}{6D_{e}C_{ab}};\tau_{R}=\frac{\rho_{S}R_{0}}{K_{S}C_{ab}}$$

(46) $$t-t_{lag}=\frac{1}{D}\left\{\left[1-3{\left(1-X\right)}^{\frac{2}{3}}+2\left(1-X\right)\right]-\left[1-3{\left(1-X_{lag}\right)}^{\frac{2}{3}}+2\left(1-X_{lag}\right)\right]\right\}\;+\frac{1}{k}\left\{\left[1-{\left(1-X\right)}^{\frac{1}{3}}\right]-\left[1-{\left(1-X_{lag}\right)}^{\frac{1}{3}}\right]\right\}$$

Li et al. [103], on the other hand, fit a Boltzman model (see Equation (47)) and a logistic model (see Equation (48)) to investigate bioleaching in gold ore heaps, concluding that the former better matches the actual oxidation effect. x is the independent variable (oxidation time) and y is the response variable (oxidation degree).

(47) $$y=A_{2}+\frac{A_{1}-A_{2}}{1+exp\left(\frac{x-x_{0}}{dx}\right)}$$

(48) $$y=A_{2}+\frac{A_{1}-A_{2}}{1+{\left(\frac{x}{x_{0}}\right)}^{p}}$$

Finally, in more recent works, Abdollahi et al. [30] modeled the kinetic of Co bioleaching considering that the chemical reaction on the particle surface controls the dissolution rate, and that diffusion through the product layer is the rate-limiting step. Laurent et al. [104] developed numerical modeling of column experiments to represent in situ bioleaching using a differential advection–reaction–dispersion model. Jalali et al. [105] used the response surface technique to model laboratory-scale column bioleaching of low-grade uranium ore using an isolate of *Acidithiobacillus ferridurans*. Zhou et al. [106], also using the response surface methodology, modeled the bioleaching of high fluorine and low sulfur uranium ore, and Sun et al. [107] optimized bioleaching parameters for high magnesium nickel sulfide ore. Li et al. [108], on the other hand, used the kinetic model controlled by surface chemical reactions or the kinetic model controlled by internal diffusion through the product layer to study the enhancement effect of sulfur on uranium bioleaching in column reactors from refractory uranium ore. Shang et al. [109] modeled the dissolution kinetic of pyrite, chalcocite, and chalcopyrite by an empirical, diffusion-like equation. Sundramurthy et al. [110] modeled the zinc bioleaching rate using a *Leptospirillum ferriphilum* isolate; the leaching data were analyzed using a shrinking core model, which revealed that the rate of leaching was inhibited by diffusion through product layer. Zhang et al. [111] developed a bioleaching of dewatered electroplating slurries for base metal extraction using an adapted microbial consortium, while that the bioleaching process dynamics was described by a modified shrinking core model, where it was established that interfacial transfer and diffusion through the solid film layer was the rate-controlling step and controlled the dissolution kinetics. Pathak et al. [112] tested different bioleaching operational strategies for the recovery of valuable metals (Ni, V, Mo, and Al) from a spent hydroprocessing catalyst using *Acidithiobacillus thiooxidans*, while the dissolution kinetic was modeled through the diffusion-controlled model and the chemically controlled model. Becci et al. [113] modeled mathematically and developed a kinetic analysis of the bioleaching of circuit boards for the extraction of copper using iron as an oxidizing agent, obtained by oxidizing Fe^2+^ through bacterial metabolism, while the models used were: an equation that describes the abundance trend of bacteria, one that represents the variation of the Fe^2+^ concentration, and a third that focuses on the extraction of copper (see Equation (49)).

(49) $$\frac{d{Cu}_{t}^{2+}}{dt}=\left[k\left({Cu}_{0}^{0}-{Cu}_{t}^{2+}\right)\left({Fe}^{3+}-m{Cu}_{t}^{2+}\right)\right]{A.W.}_{Cu}$$

where k is the rate constant given by the Arrhenius equation, ${Cu}_{0}^{0}$ is the initial Cu concentration, m is the molar ratio between the consumed Fe^3+^ and the dissolved Cu^2+^, and ${A.W.}_{Cu}$ is the Cu atomic weight.

### 4.4. Modeling of the Bioleaching Process Using Machine Learning

Although there are several authors who have studied the mineral leaching process through the use of machine learning techniques [114,115], the application to bioleaching is still an incipient area. However, in recent years, microorganism-based methodologies have been developed to recover metals from electronic waste, including bioleaching, biosorption, bioaccumulation, biotransformation, and/or biomineralization, among others [116].

Demergasso et al. [117] developed a decision support system for the bioleaching process in heaps (using the automatic learning algorithms of K means and decision trees) where a user could match the operating conditions with the historical set of data, obtaining the expected performance, such as mineral recovery, leaching agent consumption, or microbial activity. Other applications of machine learning to the mineral bioleaching process include the estimation of the recovery rate in the bioleaching process using a machine learning approach, as in the work developed by Mokarian et al. [118], where 40 regression-based machine learning algorithms were evaluated, the random forest regression being the algorithm that presented the highest performance (77% accuracy). The variables used by Mokarian et al. [118] consider the type of bacteria, temperature, pulp density, initial pH, the method used, particle size distribution, and density and type of resources, concluding that the resources, the size distribution and density of the particles, the temperature, and the type of microorganisms—bacteria and/or fungi—were the most influential variables for the estimation of the mineral recovery rate.

Although it was not directly applied to mineral recovery in bioleaching processes, Kang et al. [119] generated a model based on artificial neural networks to predict dynamic changes in the bioleaching solution, achieving accurate predictions for pH or Eh, and finding that both for the temperature and dosing, bioleaching tends to increase non-linearly. In Priyadarshini et al. [120], on the other hand, ML-based predictive models (regressions and algorithms based on random forest) were fitted to predict metal recovery from spent zinc-manganese batteries by studying the concentration of energetic substrates, pH control, temperature, and pulp density. The XGBoost model was the one that presented the best goodness of fit indicators.

More recently, and considering that metal recycling has been booming in recent years [121], some recent works have used bioleaching for recovering metals from e-waste, such as printed circuit boards (PCB) [122,123,124,125]. In order to predict the bioleaching dynamics of spent catalysts, Vyas et al. [123] used artificial neural networks to model the efficiency of Mo bioleaching from spent catalysts using microorganisms. The variation in the extraction of this metal was modeled considering the size of the particles, the density of the pulp, the temperature, and the residence time as independent variables. Annamalai et al. [125] studied the applicability of ANNs to predict the bioleaching of metals from PCB, in addition to the impact of parameters such as initial pH, pulp density, and volume as independent variables of the inoculum, while the explained variables were Ag, Cu, and Au extraction.

Along the same lines as the recycling of technological waste, and not directly applied to bioleaching dynamics, Ruhatiya et al. [124] applied an approach based on support vector regressions to optimize the bioleaching process of waste lithium ion batteries, focusing on the intermediate processes, specifically in the generation of biomass. The model generated by Ruhatiya et al. [124] presents satisfactory goodness-of-fit indicators, making it possible to report the biomass maximization for the set of independent variables sampled. Finally, Trivedi et al. [122] modeled the enzymatic bioleaching of metals from printed circuit boards (electronic waste) using RSM and IA models (for later optimization), concluding that the BBD–RSM models are statistically significant and that the models based on ANN are more accurate than those based on SVM.

## 5. Conclusions and Future Perspectives

The bioleaching outlook is encouraging as tank bioleaching technology is expected to likely increase in application for concentrates of valuable metals. The use of thermophilic and archaeal bacteria will be an important contribution, increasing leaching and metal recovery rates and allowing for the treatment of minerals such as chalcopyrite. On the other hand, space and subsurface biomining will generate more and more interest in future decades because the capabilities of microbes are expanding more and more through synthetic biology, while the prospects for bio-mining in waste mining and/or urban mining have the potential to help maximize the use of resources, supporting the move towards the circular economy.

In summary, not only bioleaching, but also biohydrometallurgy, offer the following perspectives:

- Use of deeper deposits, lower grades, and more complexity;
- Exploration of the use of space resources in situ;
- Mining of strategic metals and unconventional minerals;
- Waste mining and industrial ecology;
- Saline water processing;
- Microbe engineering;
- Removal of impurities and integrated processes;
- Development of alternative leaching;
- Use of artificial intelligence and digital twins.

Research and development in mineral bioleaching technologies is of vital importance in the current mining industry since it facilitates the economic extraction of valuable metals from very low-grade minerals which could not be commercially exploited by conventional methods. Additionally, it is an attractive alternative inside the emergence of urban mining, mining which involves the reprocessing of waste products (mainly e-waste) of modern societies. Among the advantages of bioleaching is that it does not usually use dangerous reagents, since it is a low-polluting mining and the water is recycled and volatile toxic compounds are not produced. Future work should focus on the optimization of leaching processes and their large-scale application in paradigms such as urban mining.

On the other hand, microorganisms not only have the potential to improve the biobeneficiation of minerals—the biodegradation of toxic organic compounds (waste from various industries, including mining) represents another important area of application of biological processes. An example of this is the use of certain plant species in the geological prospecting of mineral deposits, as well as the cleaning and recovery of soils contaminated with heavy metal ions.

## Figures and Tables

**Table 1:** Expressions for the conversion of particles according to the SCM model.

Expression		Broadcast on Film	Product Layer Diffusion	Chemical Reaction
constant size particles	$\mathrm{flat}\;\mathrm{plate}\;\alpha =1-\frac{e}{L}$	$\frac{t}{\tau }=\alpha \;\tau =\frac{aC_{B}L}{bkC_{A0}}$	$\frac{t}{\tau }=\alpha^{2}\;\tau =\frac{aC_{B}L^{2}}{2bD_{AB}C_{A0}}$	$\frac{t}{\tau }=\alpha \;\tau =\frac{aC_{B}L}{bkC_{A0}}$
	$\mathrm{Cylinder}\;\alpha =1-{\left(\frac{r_{n}}{r_{ext}}\right)}^{2}$	$\frac{t}{\tau }=\alpha \;\tau =\frac{aC_{B}r_{ext}}{2bkC_{A0}}$	$\frac{t}{\tau }=\alpha +\left(1-\alpha \right)\ln\left|1-\alpha \right|\;\tau =\frac{aC_{B}r_{ext}^{2}}{4bD_{AB}C_{A0}}$	$\frac{t}{\tau }=1-{\left(1-\alpha \right)}^{\frac{1}{2}}\;\tau =\frac{aC_{B}r_{ext}}{bkC_{A0}}$
	$\mathrm{Sphere}\;\alpha =1-{\left(\frac{r_{n}}{r_{ext}}\right)}^{3}$	$\frac{t}{\tau }=\alpha \;\tau =\frac{aC_{B}r_{ext}}{3bkC_{A0}}$	$\frac{t}{\tau }=1-{3\left(1-\alpha \right)}^{\frac{2}{3}}+2\left(1-\alpha \right)\;\tau =\frac{aC_{B}r_{ext}^{2}}{6bD_{AB}C_{A0}}$	$\frac{t}{\tau }=1-{\left(1-\alpha \right)}^{\frac{1}{3}}\;\tau =\frac{aC_{B}r_{ext}}{bkC_{A0}}$
Particles decrease in size with solid being dislodged	Sphere Small particles Stokes regime	$\frac{t}{\tau }=1-{\left(1-\alpha \right)}^{\frac{2}{3}}\;\tau =\frac{aC_{B}r_{ext}^{2}}{2bD_{AB}C_{A0}}$ natural convection	Does not apply	$\frac{t}{\tau }=1-{\left(1-\alpha \right)}^{\frac{1}{3}}\;\tau =\frac{aC_{B}r_{ext}}{bkC_{A0}}$
	Sphere large particles	$\frac{t}{\tau }=1-{\left(1-\alpha \right)}^{\frac{1}{2}}\;\tau =\frac{r_{ext}^{\frac{3}{2}}}{C_{A0}}=constant$ forced convection	Does not apply	$\frac{t}{\tau }=1-{\left(1-\alpha \right)}^{\frac{1}{3}}\;\tau =\frac{aC_{B}r_{ext}}{bkC_{A0}}$

## Data Availability

Not applicable.
